# Outcomes of Catheter-Based Pulmonary Artery Embolectomy in Patients With Sub-Massive to Massive Pulmonary Embolism

**DOI:** 10.7759/cureus.34877

**Published:** 2023-02-11

**Authors:** Adel Elmoghrabi, Irfan Shafi, Ahmed Abdelrahman, Heba Osman, Nouraldeen Manasrah, Mohamed Zghouzi, Adnan Halboni, Skarlet Patino, Neel N Patel, Zaher Hakim, Delair Gardi, Nasser Lakkis, M. Chadi Alraies

**Affiliations:** 1 Cardiology, Detroit Medical Center - Wayne State University, Detroit, USA; 2 Internal Medicine/Pediatrics, Detroit Medical Center - Wayne State University, Detroit, USA; 3 Internal Medicine, Detroit Medical Center, Sinai Grace Hospital - Wayne State University, Detroit, USA; 4 Internal Medicine, Detroit Medical Center - Wayne State University, Detroit, USA; 5 Internal Medicine, New York Medical College/Landmark Medical Center, Woonsocket, USA; 6 Graduate Medical Education, B J Medical College, Ahmedabad, IND

**Keywords:** flowtriever, thrombectomy, submassive pulmonary embolism, pulmonary artery thrombectomy, massive pulmonary embolism

## Abstract

Background

Pulmonary embolism (PE) is the third leading cause of cardiovascular death after myocardial infarction and stroke. The ideal therapeutic approach for these patients remains undetermined. We report our single-center outcome data for using a catheter-based pulmonary artery thrombectomy using the FlowTriever (Inari Medical, Irvine, CA) device as management for patients with submassive PE.

Methods

We retrospectively collected data from a single center of patients who underwent thrombectomy using INARI FlowTriever device. The data on baseline characteristics, procedural and clinical outcomes was collected and analysed

Results

A total of 38 patients with PE treated endovascularly with the FlowTriever device were identified: 33 with submassive PE and five with massive PE. The mean age was 65.9 years (95% CI 61.9 - 69.8), and most patients were male (73.7%). All patients had right heart strain as the main indication for thrombectomy. Four patients (10.53%) required pressor support before the procedure. In 31 patients, pre- and post-thrombectomy average mean pulmonary artery pressure (mPAP) was improved significantly by 22% (p < 0.01). Two patients had significant adverse events at 48 hours (5.26%). One patient experienced procedure-related access site hematoma and life-threatening bleeding, while another developed intraprocedural-related massive hemoptysis and cardiopulmonary arrest. Overall post-procedural length of stay was 7.7 ± 5.6 days; 52.63% of patients (n = 20) required intensive care. Three patients (7.89%) required pressor support before the procedure, and 78.9% of patients (n = 30 of 38) survived hospital discharge. Thirty patients who survived were discharged with oral anticoagulation. There were no device-related complications.

Conclusion

Randomized trials of interventional devices for submassive PE are warranted to either support or alert the medical community of the safety and efficacy of their use for patients with submassive and massive PE. In time, pulmonary embolism response team (PERT) may generate outcome data that better inform treatment decisions.

## Introduction

Pulmonary embolism (PE) is the third leading cause of cardiovascular death after myocardial infarction and stroke, with an approximately 10% 30-day mortality rate and about 100,000 deaths annually in the US [1,2]. This has gaged more interest in recent years to determine the optimal therapy for this potentially lethal disease [3].

Management strategies differ based on the severity of the disease. Patients with hemodynamic instability are defined as massive PE that requires lifesaving reperfusion therapy. Asymptomatic patients with hemodynamic stability and lack of right ventricular (RV) dysfunction, normal biomarkers, or low simplified Pulmonary Embolism Severity Index (sPESI) scores have a relatively benign outcome and represent a low-risk category [2].

A more controversial group remains in between this spectrum, defined as patients with intermediate-risk or submassive PE that exhibit biochemical evidence of myocardial injury and or imaging findings of RV dysfunction. This group represents 35% to 55% of hospitalized patients with PE [4]. The 7-30-day mortality for intermediate-risk PE managed with anticoagulation alone is reported at 2-3% in randomized control trials, although observational cohorts have identified mortality rates of up to 15% on 7-90-day follow-up with approximately 10% of the patients decompensating to a high-risk category [4,5].

The adverse outcomes of this population on anticoagulation therapy have prompted physicians to consider therapeutic escalation strategies, whether medical, catheter-based therapy (CBT), or surgical embolectomy. The ideal therapeutic approach for these patients remains undetermined.

We, at this moment, report our single-center outcome data for using a catheter-based pulmonary artery thrombectomy using the FlowTriever device as management for patients with submassive PE.

## Materials and methods

We retrospectively identified the patients with submassive and massive pulmonary embolisms in a single hospital system who underwent catheter-directed thrombectomy using the INARI FlowTriever device. The baseline demographics and comorbidities were collected from the hospital chart review. Data on procedural outcomes, such as pulmonary artery (PA) pressure, hemoglobin (Hgb) level, systolic blood pressure (SBP) readings, and heart rate (HR) readings before and after the thrombectomy by FlowTriever were collected and analyzed.

Our primary clinical outcome was 48-hour mortality and 30-day mortality, while secondary outcomes included acute kidney injury (AKI), need for vasopressor support, deep venous thrombosis (DVT), major bleeding, and percentage of ICU admissions. All the data on baseline comorbidities, procedural outcomes, and primary and secondary clinical outcomes were tabulated and analyzed using Microsoft Excel (Microsoft® Corp., Redmond, WA).

## Results

Thirty-eight patients with PE treated endo-vascularly with the FlowTriever device were identified: thirty-three with sub-massive PE and five with massive PE. Thirty-three patients had an sPESI score ≥ 1. The mean age was 65.9 years (95% CI 61.9 - 69.8), and most patients were male (73.7%). 18.42% of patients had chronic kidney disease (CKD), 13.16% of patients had a history of coronary artery disease (CAD), 63.14% of patients had hypertension, and 50% of patients were found to have concomitant DVT. 13.2% of patients had a history of malignancy, 31.6% of patients were obese, and 15.8% were found to be positive for COVID-19 on admission. Additional patient demographics and PE-relevant variables are outlined in Table 1. All patients had right heart strain as the main indication for thrombectomy. Four patients (10.53%) required pressor support before the procedure (Table 1).

**Table 1:** Baseline characteristics of the study population CKD: Chronic Kidney Disease, COPD: Chronic Obstructive Lung Disease, CHF: Congestive Heart Failure, CAD: Coronary Artery Disease, CVA: Cerebrovascular Accident, SLE: Systemic Lupus Erythematosus, PAD: Peripheral Arterial Disease, HTN: Hypertension, DM: Diabetes Mellitus, TPA: Tissue Plasminogen Activator.

Baseline Characteristics	n (%) or mean ± SD
Age	65.9 ± 11.7
Female	10 (26.32)
CKD	7 (18.42)
COPD	7 (18.42)
CHF	3 (10.53)
CAD	5 (13.16)
Prior CVA	4 (10.53)
SLE	1 (2.63)
PAD	1 (2.63)
Obesity	12 (31.58)
HTN	24 (63.14)
DM	9 (23.68)
COVID-19	6 (15.79)
Malignancy	5 (13.16)
Smoking	7 (18.42)
Contraindication to TPA	6 (15.78)
Pressor Support Prior to INARI	4 (10.53)
IV anticoagulation before INARI	34 (89.47)

The most common FlowTriever device sizes used were 20 and 24 Fr (n = 35). In 31 patients, pre- and post-thrombectomy average mean pulmonary artery pressure (mPAP) was improved significantly by 22% (p < 0.01). Mean PA pressure before and after thrombectomy was 35.09 mmHg (95% CI 32.2 - 37.9) and 28.51 mmHg (95% CI 25.7 - 31.3), respectively. The mean hemoglobin before using the FlowTriever device was 12.44 g/dl (95% CI 11.5 - 13.38), and the mean hemoglobin after was 10.89 g/dl (95% CI 9.9 - 11.84) with a statistically significant mean difference of 1.55 (95% CI 1.07 - 2.04), p < 0.01. The mean SBP before thrombectomy was 127 mmHg (95% CI 120 - 134), and the mean SBP after was 124 mmHg (95% CI 117 - 131). The mean HR before was 95 (95% CI 87 - 104), and the mean HR after INARI was 92 (95% CI 83 - 101) (Table 2).

**Table 2:** Procedural outcomes before and after thrombectomy using FlowTriever

Outcomes	Before FlowTriever	After FlowTriever
Mean PA pressure (mmHg)	35.09 (95% CI 32.2 - 37.9)	28.51 (95% CI 25.7 - 31.3)
Mean Hgb (g/dl)	12.44 (95% CI 11.5 - 13.38)	10.89 (95% CI 9.9 - 11.84)
Mean SBP (mmHg)	127 (95% CI 120 - 134)	124 (95% CI 117 - 131)
Mean HR (bpm)	95 (95% CI 87 - 104)	92 (95% CI 83 - 101)

**Figure 1:**
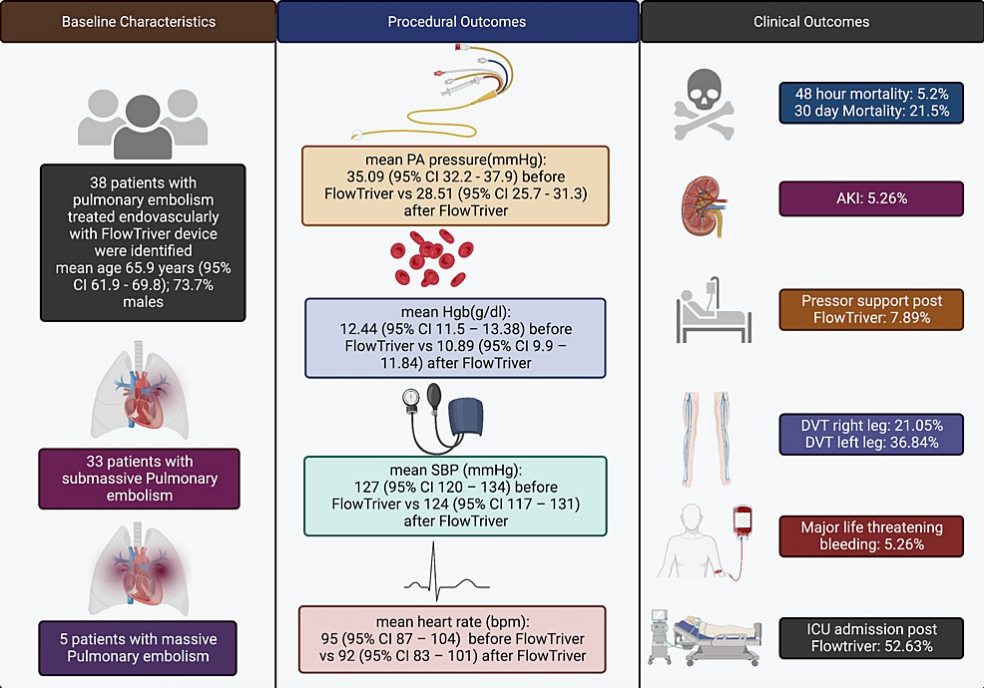
Visual abstract showing baseline characteristics, procedural and clinical outcomes Created using Biorender.com PA: Pulmonary artery; SBP: Systolic blood pressure; AKI: Acute kidney injury; DVT: Deep venous thrombosis.

Two patients had significant adverse events at 48 hours (5.26%). One patient experienced procedure-related access site hematoma and life-threatening bleeding, while another developed intra-procedural-related massive hemoptysis and cardiopulmonary arrest. Overall post-procedural length of stay was 7.7 ± 5.6 days. 52.63 patients (n = 20) required intensive care. Three patients (7.89%) required pressor support before the procedure. 78.9% of patients (n = 30 of 38) survived hospital discharge. Thirty patients who survived were discharged with oral anticoagulation. There were no device-related complications (Table 3) (Figure 1).

**Table 3:** Clinical outcomes of the FlowTriever device AKI: Acute Kidney Injury, MAE: Major Adverse Event, DVT: Deep Venous Thrombosis.

Outcomes	n (%)
Mortality	8 (21.05)
AKI	2 (5.26)
Pressor support Post INARI	3 (7.89)
MAE within 48 hrs	2 (5.26)
Major life-threatening bleeding	2 (5.26)
ICU admission Post INARI	20 (52.63)
DVT Right Leg	8 (21.05)
DVT Left Leg	14 (36.84)

## Discussion

The lack of a single optimal treatment for sub-massive PE and the paucity of data on innovative alternatives is immense. With the advent of percutaneous intervention in acute coronary syndromes and acute stroke, little comparative research has focused on novel therapies to decrease morbidity and mortality in patients with PE. Hence, there has been growing interest in novel endo-vascular therapies, in addition to the advent of pulmonary embolism response teams [4]. This would serve to improve VQ mismatch by enhancing perfusion, decreasing RV afterload by decreasing pulmonary vascular resistance (PVR), and subsequently improving left ventricular (LV) preload. The rationale for the use of these devices is to deter possible hemodynamic collapse and death resulting from developing right-sided heart failure and from expediting symptom resolution. Importantly, however, to date, no prospective study has demonstrated a mortality benefit associated with the use of any interventional therapy in any population of patients with PE, granted it is difficult powering trials for these clinically important outcomes. It would require 1500 to 2000 patients to adequately power randomized trials to demonstrate a mortality benefit and superiority over a short time [4].

Other potential but unproven benefits include the prevention of recurrent PE by reducing thrombotic burden and prevention of chronic thromboembolic pulmonary hypertension (CTEPH) and preservation of the normal hemodynamic response to exercise over the long term [4].

The safety and efficacy of endo-vascular interventional therapy for acute PE and determining which patients with PE would derive the most significant net benefit from their use in various clinical settings is warranted.

The PEITHO trial revealed decreased cardiac decompensation in patients with intermediate-risk PE receiving thrombolytic therapy compared to medical therapy but at the expensed rate of major bleeding including intracranial bleeding [2,5,6].

To mitigate this risk, interest in endovascular therapies evolved, which aimed at improving clinical and hemodynamic parameters as a result of reducing vascular occlusion. The PERFECT multi-center registry assessed reduced doses of catheter-directed thrombolysis and fragmentation in comparison to tissue plasminogen activator (TPA) which resulted in a significant reduction in pulmonary artery systolic pressure (PASP) at an attenuated bleeding risk although not completely eradicated [7].

Endovascular catheter-directed ultrasound and thrombolysis studies such as SEATTLE II and ULTIMA were also associated with a reduction in RV/LV ratios and pulmonary artery systolic pressures in comparison to heparin yet with a continued risk of major bleeding of up to 10% in the Seattle II trial [8,9].

Sole mechanical thrombectomy, which initially involved pure fragmentation of the thrombus, decreased global pulmonary vascular resistance and RV afterload, however, increasing the risk of chronic thromboembolic pulmonary hypertension from distal embolization of fragmented thrombi [10]. Moreover, the results of small studies suggested hemodynamic deterioration with such procedures [2].

Aspiration thrombectomy via large bore cannulas was developed aiming to debulk large proximal PE circumventing the initial attempts at manual aspiration thrombectomy of enormous thrombus burden via small diameter catheters. The AngioVac system (AngioDynamics, Latham, NY) gained little success due to its rigidity and lack of maneuverability through the RV and pulmonary arteries, in addition to high rates of hemodynamic decompensation and cardiac complications.

The AngioJet system (Boston Scientific, Maple Grove, MN) combining both a pharmacy mechanical approach via catheter-directed thrombolysis followed by aspiration thrombectomy showed promising clinical results although advertently associated with arrhythmia, hypotension, and hemoglobinuria and in rare cases with kidney failure [11]. The FlowTriever System (Inari Medical, Irvine, CA) is one of the latest percutaneous aspiration thrombectomy catheters developed.

The safety of CBT has been a major area of interest to elucidate further the benefits versus risks of this therapeutic strategy to manage PE. Thrombectomy devices such as FlowTriever are uniformly larger catheters and advanced over stiffer wires than used for catheter-directed thrombolysis and theoretically impose a higher risk of intra-procedural complications. Most available data on CBT is based on small single-arm trials and hence we opted to report our initial center experience regarding the safety and rate of complications.

The multicenter prospective Flare trial assessed the risk of major adverse events (MAEs) to determine the safety of this device. The MAEs evaluated in prior CBT studies and reported in the MAUDE (Manufacturer and User Facility Device Experience) database include mortality, major bleeding, rates of intracranial hemorrhage, and hemodynamic and respiratory decompensation.

Similar to the Flare study, the overall rate of patients developing MAE reported in our study was 5%. Two patients developed major bleeding based on the Valve Academic Research Consortium (VARC-2) criteria. One patient developed a procedure-related pulmonary complication with massive hemoptysis resulting in hemodynamic and respiratory decompensation resulting in on-table demise. It is difficult to discern if this was a device-related pulmonary complication or a result of a wire-related injury or pulmonary infarction due to distal embolization as a result of thrombus fragmentation. The MAUDE database reported four additional cases of acute pulmonary hemorrhage with the use of the FlowTriever device.

We report one case of access site major bleeding as a result of access site hematoma in addition to suspected retroperitoneal hematoma and subsequent demise. To note, this patient had central venous access and arterial line placement due to obstructive shock on the ipsilateral side of access prior to undergoing CBT. Thus, her potential for vascular-related complications is confounded by prior procedures.

No intracranial hemorrhages were reported in our study which resonates the low incidence of such complications with catheter-based thrombectomy in comparison to thrombolytic therapy and catheter-directed thrombolysis.

There is heterogeneity in the definition of major bleeding in the literature and widely varied in different studies of CBT for pulmonary embolism.

The Flare study used the VARC-2 classification to define bleeding events which we opted to use in our study. One episode of major bleeding was reported in this study (0.9%) due to intra-procedural pulmonary hemorrhage, likely due to pulmonary infarct and reperfusion injury, as proven on surgical pathology. In our experience, there were at least two fatal bleeds (5%) representing a significantly higher rate of bleeding secondary to a post-procedural presumed retroperitoneal hematoma and massive intra-procedural hemoptysis. It is difficult to adjudicate if our patient with intra-procedural pulmonary hemorrhage was a result of a procedure or device-related complication [3].

Furthermore, although there were no other cases of overt bleeding that resulted in >3g drop in hemoglobin or required blood transfusion to meet VARC criteria for life-threatening/major bleeding, our study did reveal a statistically significant post-procedural decrease in hemoglobin counts.

If patients who had more than 2gm drop in hemoglobin post-procedurally are accounted for as in other studies of CBT for PE such as ULTIMA and OPTALYSE an additional 10/38 patients would be included resulting in a total of 31% rate of major bleeding. This underscores the significant blood loss with the use of large bore catheter-based thrombectomies such as with the FlowTriever device. This is a substantially higher degree of major bleeding in comparison to other CBT studies with the highest rate of major bleeds reported being 4% in the OPTALYSE study.

This can be explained by the fact that other catheter-based therapies use substantially smaller-sized catheters and hence less procedural blood loss. Another FDA-approved CBT for the management of PE such as the AngioJet rheolytic thrombectomy (ART) had similar major bleeding rates as reported in our study although incorporating all patients with >2 gm hemoglobin drop [12].

Das et al. reported procedure-related anemia (mean hemoglobin drop of 0.49 g/dl) as the only significant minor complication using ART with no bleeding complications and no patients requiring transfusion [11].

Many of the patients in our study had preexisting anemia of chronic disease at baseline. The impact/outcome of this superimposed oxygen-losing capacity due to procedure-related acute blood loss anemia in the patients preexisting VQ mismatch is unknown.

AngioVac which uses a comparable catheter size to the flow retriever uses an extracorporeal circuit that recirculates the blood through a filter via a second percutaneously placed re-infusion venous cannula, hence minimizing blood loss.

The significant blood loss reported in our study was not highlighted in the Flare study, however, was noted in another large single-center experience using the FlowTriever device where aspiration-related blood loss averaged 280 mL per case, with a maximum single case aspiration-related blood loss of 520 mL. Hematocrit dropped on average by 4.6% ± 2.8% per case. There was only one patient requiring blood transfusion and the study did not comment on major bleeding. They did note a maximum single case of blood loss was attributed to the operator’s initial use of the device and decreased with operator experience [13].

Another study by Graif et al. evaluated thrombectomy with the INARI device and revealed an approximate reduction of 11.5% in hemoglobin, which was not significantly different than the reduction in the catheter-directed thrombolysis group although they did not comment if this was a statistically significant hemoglobin drop pre- and post-procedurally. Comparatively, our cohort had a mean difference of 1.55 g/dL pre- and post-FlowTriever use which is a similar result to both studies by Graif et al. and Wible et al. [13,14].

To circumvent this, filtering systems for auto-transfusing aspirated blood after thrombus filtration have been developed and introduced to complement future procedures. The overall outcome of this procedural modification on post-procedural hemoglobin counts is not reported in our experience however would be expected to attenuate the significant hemoglobin loss noted in our study.

To further illustrate the bleeding complications of CBT, future studies should use the universal BARC (Bleeding Academic Research Consortium) definitions which is a well-validated tool that allows comparison with many prior interventional trials across various disease processes and unifies outcomes reported.

On the contrary, patients with preexisting contraindications to lytic therapy or heparin who are at high risk for bleeding, such as post-operative patients, who represent about 30% of the PE population have limited therapeutic options. 3CBT serves as a solitary therapeutic strategy in this critically ill population. Fifteen percent of patients in our study had contraindications to lytic therapy. Bunc et al. studied 25 patients with high-risk PE and contraindications to lytic therapy undergoing CBT which proved successful in 80% of patients with 68% surviving to discharge [15].

Successful and safe percutaneous extirpation of clot in transit has also been reported as a revolutionary treatment using the FlowTriever device [16].

The main short-term surrogate endpoints evaluated in CBT trials and studies are improvement in RV function, reduction in PA systolic pressure [13], and decreased angiographic thrombotic burden. Our study did reiterate this with a statistically significant improvement in mean pulmonary artery pressure before and after the FlowTriever-based thrombectomy. Previous studies have coupled this finding with a decreased RV LV ratio after CBT in comparison to medical therapy. It is hypothesized that given increased RV/LV ratio has been independently associated with mortality at 30 days, a reduction in this ratio via interventional therapy may result in decreased mortality [3].

We were unable to directly compare this parameter in our study due to the lack of available imaging data using the same modality pre- and post-FlowTriever use [4].

As noted in the Flare study, there was no statistically significant difference in vital signs pre- and post-FlowTriever device.

The rates of acute kidney injury were 5% in our study which is considerably less than reported in AngioJet rheolytic thrombectomy which was up to 39% in some studies [12]. Other complications such as transient bradycardia, AV block were not seen in our study which are believed to be secondary to hemolysis followed by adenosine release occurring at activation bursts during rheolytic thrombectomy. The occurrence of severe hyperkalemia and hemoglobinuria was also not observed in our study.

The 48-hour mortality in our study was 5% which is considerably higher than reported in the Flare trial. In another retrospective review of CBT with catheter-directed thrombolysis versus large bore thrombectomy with the FlowTriever device, there were two reported procedure-related mortalities representing 7.7% of their sample although not definitively attributed to the device which had yet to be inserted into the patient’s body at the time of the arrest [14].

The discrepancy between quoted mortality rates in RCTs and in observational data such as ours is that RCTs enroll selected populations which exclude patients with significant concomitant comorbidities. For example, in the Flare trial patients with hemodynamic instability or high-risk PE were excluded from the trials. On the contrary, patients reported in our study had higher sPESI score reflecting an overall sicker group of patients. Subsequently, this also explains the increased intensive care length of stay reported in our study.

There were two procedure-related mortalities in our study at 48 hours. Another six patients died during the 30-day follow-up due to comorbid conditions. One patient died presumably due to hemodynamic deterioration from a massive PE.

Limitations

There are some limitations of our study which are worth noting. Firstly, the study was retrospective in nature, which can potentially include confounding data due to subjective variations. Secondly, the study population was small and from a single center, which can potentially have selection bias and that may limit the generalizability of our outcomes and detection of less common potential outcomes/complications. Third, the study population was non-randomized and our study did not include a control group or comparator arm. Fourth, we did not have post-procedure echocardiographic and ECG data available for all the patients, which limited our ability to report outcomes such as change in RV strain or change in RV/LV ratio post procedure.

## Conclusions

The study population included a mix of patients with massive and submassive PE, which may serve as a confounding factor for the treatment effect and complication rates. MAE in patients undergoing CBT with the FlowTriever device is low but not negligible. Further studies of CBT are needed to maximize generalizability. The rates of significant blood loss considered as major bleeding by various definitions is substantial with the FlowTriever device. This undermines the importance of considering autologous transfusion of aspirated blood. Randomized trials of interventional devices for submassive PE are warranted to either support or alert the medical community of the safety and efficacy of their use for patients with submassive and massive PE. The long-term follow-up of such interventions will also determine the effectiveness in preventing sequala of pulmonary embolism such as chronic thromboembolic pulmonary hypertension and research into the comparative effectiveness of these devices and interventions may take the form of clinical trials or observational outcomes research and cost-effectiveness studies. In time, pulmonary embolism response teams (PERTs) may generate outcome data that better inform treatment decisions.
